# Study and Characterization of Regenerated Hard Foam Prepared by Polyol Hydrolysis of Waste Polyurethane

**DOI:** 10.3390/polym15061445

**Published:** 2023-03-14

**Authors:** Xiaohua Gu, Xiaoyao Wang, Xinyu Guo, Siwen Liu, Qi Li, Yan Liu

**Affiliations:** 1 School of Energy and Building Environment of Guilin University of Aerospace Technology, Guilin 541004, China; 2 School of Material Science and Engineering of Qiqihar University, Qiqihar 161006, China; 3 State Key Laboratory for Modification of Chemical Fibers and Polymer Materials, College of Materials Science and Engineering, Donghua University, Shanghai 201620, China; 4 College of Chemistry and Chemical Engineering of Qiqihar University, Qiqihar 161006, China; 5 College of Innovative Material & Energy, Hubei University, Wuhan 430062, China; 6 Fishery College, Zhejiang Ocean University, Zhoushan 316022, China

**Keywords:** waste polyurethane elastomer, degradation and recovery, glycolysis, diols, regenerated polyurethane foam

## Abstract

In this paper, four different kinds of diols were used for the alcoholysis of waste thermoplastic polyurethane elastomers. The recycled polyether polyols were used to prepare regenerated thermosetting polyurethane rigid foam through one-step foaming. We used four different kinds of alcoholysis agents, according to different proportions of the complex, and we combined them with an alkali metal catalyst (KOH) to trigger the catalytic cleavage of the carbamate bonds in the waste polyurethane elastomers. The effects of the different types and different chain lengths of the alcoholysis agents on the degradation of the waste polyurethane elastomers and the preparation of regenerated polyurethane rigid foam were studied. Based on the viscosity, GPC, FT-IR, foaming time and compression strength, water absorption, TG, apparent density, and thermal conductivity of the recycled polyurethane foam, eight groups of optimal components were selected and discussed. The results showed that the viscosity of the recovered biodegradable materials was between 485 and 1200 mPa·s. The hard foam of the regenerated polyurethane was prepared using biodegradable materials instead of commercially available polyether polyols, and its compressive strength was between 0.131 and 0.176 MPa. The water absorption rate ranged from 0.7265 to 1.9923%. The apparent density of the foam was between 0.0303 and 0.0403 kg/m^3^. The thermal conductivity ranged from 0.0151 to 0.0202 W/(m·K). A large number of experimental results showed that the degradation of the waste polyurethane elastomers by the alcoholysis agents was successful. The thermoplastic polyurethane elastomers can not only be reconstructed, but they can also be degraded by alcoholysis to produce regenerated polyurethane rigid foam.

## 1. Introduction

Plastic is an indispensable material in our lives [1,2]. As a part of plastics, polyurethane (PU) has been rapidly developed in household goods, the construction industry, daily necessities, the transportation industry, household appliances, and other fields since its emergence [3,4,5]. PU can be used to prepare soft polyurethane foam, hard polyurethane foam, polyurethane elastomers, coatings, adhesives, and other products [6] which are deeply loved by people. Thermoplastic polyurethane elastomers, with their excellent properties and wide application, have become important thermoplastic elastomer materials whose molecules are largely linear, with no or little chemical cross-linking [7,8]. There are many physical cross-links formed by hydrogen bonds between linear polyurethane molecular chains, which strengthen the material’s form and endow it with many excellent properties, such as a high modulus, high strength, and excellent wear resistance, chemical resistance, hydrolysis resistance, high and low temperature resistance, and mold resistance [9,10,11,12,13,14]. With the wide application of polyurethane elastomers in various industries, the global production and consumption of polyurethane have been increasing year over year at an amazing speed [15]. At the same time, under such tremendous consumption, polyurethane elastomer waste has caused serious environmental pollution and resource waste. With the implementation of the global concept of environmental protection and sustainable development, the recycling and sustainable utilization of polyurethane waste has become a global issue [16].

For the recycling and sustainable development of polyurethane waste, landfill and incineration were primarily used in the beginning of its development, but the pollution of land caused by landfill is immeasurable. Although incineration is better than a landfill as it does not occupy land resources, the carbon dioxide and other gases generated by incineration cause a serious burden on the atmospheric protective layer [17,18], and people’s awareness of environmental protection and legal restrictions relating to polyurethane waste have caused its disposal via landfills and incineration to be banned. Due to its three-dimensional network structure [19], in recent years, various recycling methods for polyurethane waste have been studied, including the mechanical method, pyrolysis method, microbial method, and chemical recovery method [20,21]. Among the many recycling methods, chemical recycling can effectively break the carbamate bond in the chain segment of polyurethane waste so as to obtain valuable small molecular monomers for sustainable use [22,23]. Therefore, this method has been recognized by many scholars.

Among the many chemical recovery methods, alcoholysis has been favored for advantages such as green environmental protection, low energy consumption, less impurities in the recycled materials, and directional bond-breaking by catalysts [24,25]. In this paper, different types of small molecule diols were used for the combination ratio to study the degradation and recycling of used polyurethane elastomers. Alcoholysis agents with different ratios successfully degraded the used polyurethane elastomers into small molecule alcohols, and recycled polyurethane rigid foam was successfully prepared by using the small molecule alcohols obtained from the degradation and recovery of the elastomers. The effects of different types of alcoholysis agents on the degradation of polyurethane elastomers were studied, and they have provided certain reference significance for the selection of alcohols and the combination of two components in the degradation of polyurethane elastomers.

## 2. Materials and Methods

### 2.1. Materials and Reagents

The reagents used in this experiment are shown in Table 1.

The equipment used in this experiment is shown in Table 2.

### 2.2. Preparation of the Titanium Nanosystem Catalysts

**Figure 1:**
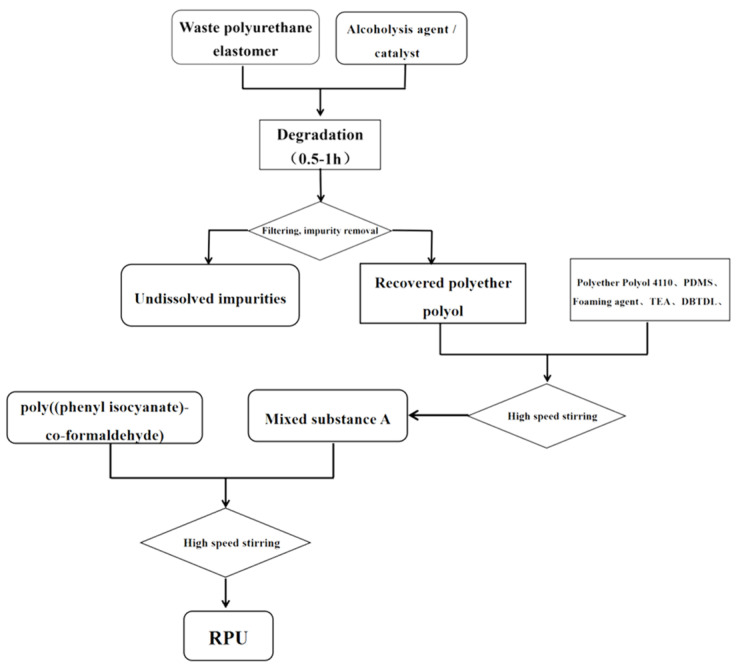
Experimental flow chart.

In this experiment, four different alcoholysis agents were selected: butanediol, propylene glycol, monodiethylene glycol, and ethylene glycol. Through the previous cumulative work in the laboratory, the four alcoholysis agents were paired to form a two-component alcoholysis agent, and the alcoholysis reaction was carried out on the waste polyurethane elastomer. The specific ratios of the alcoholysis agents were: I = EG:PG (A1–A5); II = EG:BDO (B1–B5); III = PG:BDO (C1–C5); IV = DEG:BDO (D1–D5); V = DEG:EG (E1–E5), and VI = DEG:PG (F1–F5). The mass ratios of the alcoholysis agents were 40:40, 30:50, 50:30, 60:20, and 20:60. Based on the above experimental design, the degradation steps for the waste polyurethane elastomers were as follows (the process is also shown in Figure 1):

1. The waste polyurethane elastomer was cut and processed to 5–10 mm small sections.
2. We mixed 80 g small molecular alcohol and catalyst (KOH) and added them into a 1000 mL spherical reactor. We then placed the reactor in a heating jacket and stirred to 90 °C until the catalyst was completely dissolved.
3. We added 80 g of chopped waste polyurethane elastomer into the reactor and heated it up to 180 °C. At that time, we accelerated the stirring speed and waited for the polyurethane elastomer to dissolve, gently stirring for 0.5–1 h.
4. We stopped the reaction and cooled the mixture to room temperature before pouring it into a disposable plastic cup.
5. The hydroxyl value and viscosity of the sample were measured, and the foaming experiment was carried out after the value had reached the standard.
6. We took a certain amount of alcoholysis products and added the catalyst, foaming agent, and surfactant, stirring the mixture evenly. Then, we added polyphenyl polymethylene polyisocyanate (PAPI) at a ratio of 1:1 and immediately stirred with a high-speed mixer for 10 s until the mixture was uniform, and we waited for the foam to slowly form.
7. The sample preparation and testing could then be carried out on the regenerated polyurethane hard foam, which was to be prepared after curing and cross-linking for 24 h.

### 2.3. Performance Test and Structural Characterization of the Polyurethane Rigid Foam

Viscosity analysis: An NDJ-5S digital viscometer was used for the viscosity testing. A number of degradation products were taken and placed in the container and a suitable rotor was selected, as well as a suitable rotational speed, and the viscosity testing was carried out at 25 °C.

Compression strength test: With reference to the GB/T8813-2008 test standard, we selected a sample size of 50 mm × 50 mm × 50 mm and we used an EFS-24RE universal testing machine for the compression strength test.

Water absorption test: With reference to the GB/T8810-1988 test standard, the sample size was 50 mm × 10 mm × 10 mm, and we used distilled water to determine the water absorption, weighing the mass of the sample before and after immersion, and then calculated its water absorption.

Hydroxyl value determination: With reference to the GB/T12008.3-2009 standard, we took an appropriate amount of oligomeric regenerated polyether polyol in 10 mL conical flasks and we used ester anhydride method—pyridine for the hydroxyl value determination.

Thermal conductivity analysis: With reference to the QB/T3806-1999 standard, the sample size was 200 mm × 200 mm × 20 mm, and we used a FEHC-S thermal conductivity tester acquired from Changzhou Hua’ao Instrument Manufacturing Co., Changzhou, China.

SEM analysis: We cut the regenerated polyurethane rigid foam specimens into appropriate thin slices (we did not squeeze them), and then we used SEM to observe the microstructures of the bubble pores of the foam (magnified 20 times) and identify the clear, complete, and uniform areas of the bubble pore structures for observation. We recorded the observation results.

FT-IR test: A GR-285 IR produced by Dalian Precision Scientific Instruments Co., Ltd., Dalian, China was used to analyze the structures of the foam samples. The samples were made using the KBr pressing method, and the test wavelength range was 500–4000 cm^−1^.

TG analysis: TG analysis was performed by using TG under a nitrogen atmosphere at a rate of 30 °C/min, from room temperature (25 °C) to 500 °C. The gas flow rate was selected to be 50 mL/min, the carrier gas was air, and alumina was used as a reference object.

Determination of molecular weight distribution coefficient: The molecular weight distribution of the regenerated polyether polyol was determined by gel permeation chromatography (GPC). The measurements were performed using a thermal scientific chromatograph equipped with an isocratic DionexUltra3000 pump and a RefrtoMax521 refractive index detector. The separations were performed in four Phenomenex Phenogel GPC columns with a separation temperature of 30 °C, a particle size of 5 µm, and porosities of 105, 103, 102, and 50, respectively, located in an ultimate thermostatic column at 3000 °C. The mobile phase was tetrahydrofuran (THF) at a flow rate of 1 mL·min^−1^. The samples were first dissolved with N, N—Dimethylformamide (DMF), followed by 1.6 wt.% THF, and then filtered through a nylon filter with a pore size of 2 mm and prepared for use.

## 3. Results and Discussion

### 3.1. Degradation Mechanism of the Waste Polyurethane Elastomers

Under the action of the two-component alcohols and catalysts, the carbamate in the chain segment of the polyurethane elastomer was broken and replaced by small molecular alcohol chains to generate polyether polyols [26,27]. The main reaction is:

R_1_-NHCOO-R_2_ + HO-R_3_-OH → R_1_-NHCOO-R_3_-OH + R_2_-OH (1)

R_1_-NHCOO-R_3_-OH → R_1_-NH-R_3_-OH + CO_2_ (2)

Under the condition of a high temperature reaction, there will be many groups involved in the reaction, and many side reactions will occur. The primary side reactions are the urea group fracture in the alcoholysis agent, which generates small molecules of amines and small molecules of alcohols [28]. The specific reactions are as follows:

R_1_-NHCONH-R_2_ + HO-R_3_-OH → R_1_-NHCOO-R_3_-OH + R_2_-NH_2_ (3)

**Figure 2:**
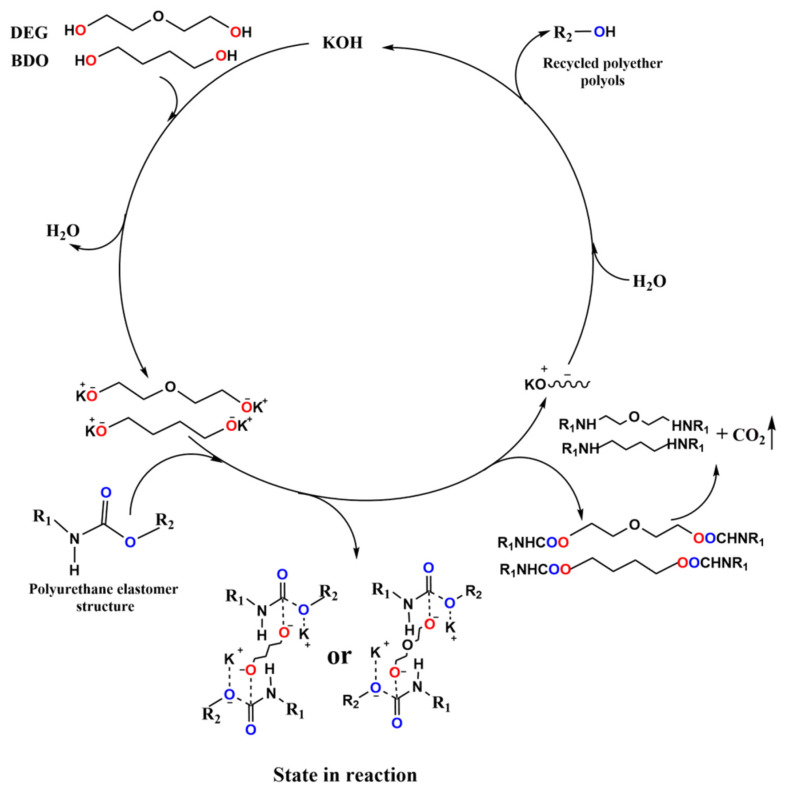
Catalytic degradation mechanism of the used polyurethane elastomer.

Taking DEG and BDO alcoholysis agents as an example, under the catalysis of an alkaline catalyst (KOH) at a high temperature in the reaction reactor, the used polyurethane elastomer and the alcoholysis agent broke and were replaced by the alcoholysis agent, thus generating polyether polyols [29]. The specific reaction mechanism is shown in the following (Figure 2):

### 3.2. Small Molecular Alcohol Is Degraded and Foamed

The purpose of this study was to explore the route of industrialization such that different types of two-component alcohols could be compared so as to select the optimal ratio of alcoholysis agents based on the viscosity of the degradation material, the compression strength, the thermal conductivity of the prepared regenerated polyurethane rigid foam, and the properties of the milky white time and gel time in the foam. According to the experimental data, the data corresponding to the eight groups of optimal components are shown in Table 3 and Table 4.

### 3.3. FT-IR Analysis of the Degradation Materials

**Figure 3:**
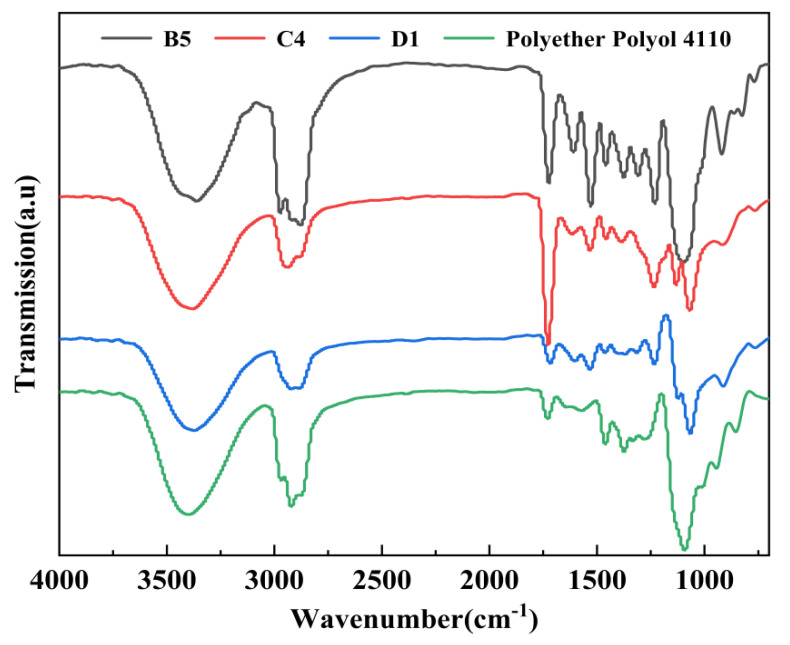
Infrared spectra of B5, C4, and D1 degradation products and polyether 4110.

Three groups (B5, C4, and D1) were selected from the eight optimal components. FT-IR was used to identify the degradation products in the glycolysis process, and the infrared spectroscopy was compared with commercially available polyether polyol 4110. The results are shown in Figure 3.

Compared with commercially available polyether 4110, the degradation products of B5, C4, and D1 had stronger absorption bands within 3500–3300 cm^−1^, which are the stretching vibration peaks of the alcohol hydroxyl group (-OH) [30]. There was a strong absorption band near 1732–1708 cm^−1^, which is a benzene-type flood frequency peak [31]. At 1054 cm^−1^, there was a clear strong absorption band, which was the polyether polyurethane ether group (-O-) absorption band, and this is the characteristic peak of polyether polyol [32,33], which is similar to the characteristic peak of commercially available polyether polyol. It can be concluded that the degradation product was the mixed product of polyether polyol and aromatic polyol. Therefore, the glycolysis product was expected to be a substitute for commercially available polyether 4110 for the preparation of the regenerated polyurethane foam.

### 3.4. Viscosity of Degradation Products

Polyether polyols are one of the most important raw materials for the synthesis of polyurethane foam, and their properties have attracted extensive attention. Their main properties are viscosity, hydroxyl value, acid value, etc. The viscosity of polyether polyols can be intuitively observed and is reflected in the differences in their fluidity. The synthesis of general polyurethane foam is optimized for polyether polyols with low viscosity and good fluidity. In this paper, the viscosity of all the degradation materials prepared by alcoholysis was less than 1700 mPa·s, while the viscosity of commercially available polyether 4110 is 4300–4500 mPa·s. Therefore, it could be seen that the degradation materials obtained by the alcoholysis degradation of waste polyurethane elastomers had excellent fluidity. The viscosity of the eight groups of degradation materials selected by the performance test of the degradation materials and the recycled polyurethane foam ranged from 400 to 1200 mPa·s. For the degradation materials recovered from the same waste polyurethane elastomer, the viscosity span was large because the alcoholysis agents used were different. Under the condition of the same catalyst, raw materials, and experimental conditions, the only element that may have affected the performance of the degradation materials was the difference in the type and proportion of alcoholysis agent used in the process of alcoholysis [34]. The length of the chain segment and the number and type of functional groups (-OH, -O-) in the alcoholysis agent used are all important indicators that will affect the final viscosity [35].

**Figure 4:**
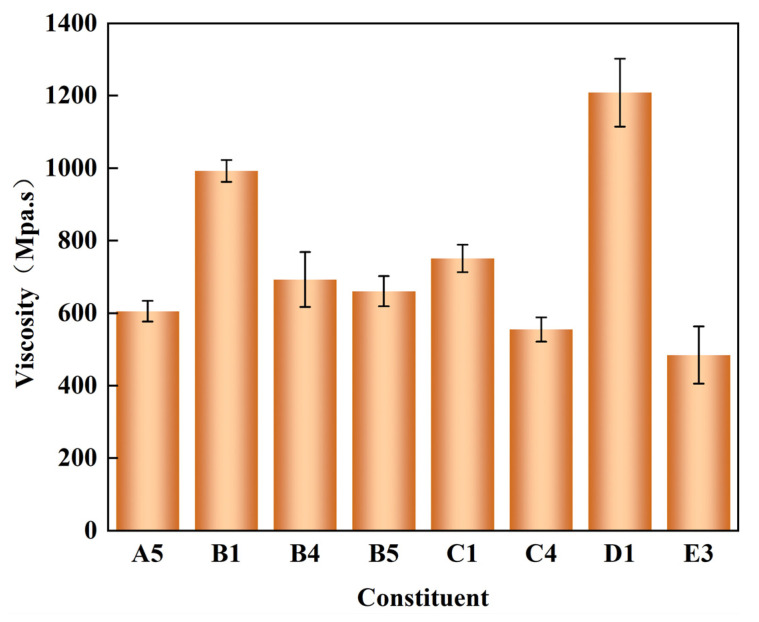
Determination of the viscosity of the degradation products of the eight groups of optimal components.

It can be clearly seen from the experimental data (Figure 4) that a longer chain segment and more functional groups (-OH, -O-) led to a greater level of viscosity in the degradation material obtained after alcoholysis. This is because the longer the chain segment of the small molecule alcohols was obtained after the carbamate in the chain segment of the waste polyurethane elastomer was broken and reacted with the alcoholysis agent at high temperature due to the longer the chain segment of the alcoholysis agent. Therefore, the viscosity of the resulting degradation product would also increase. The higher the number of functional groups of the alcoholysis agent, the denser the cross-linked network structure of the carbamate grafted onto the alcoholysis chain segment after fracture, and thus, the viscosity of the degradation product increased.

### 3.5. GPC Determination of the Degradation Products

In this experiment, under the catalysis of an alkaline metal catalyst (KOH), the carbamate bond of the waste polyurethane elastomer gradually broke at a high temperature and was replaced by alcoholysis agents, forming into small molecule polyether polyols. The side reaction at high temperatures generated amines, TDA, and other substances. Due to the poor solubility of the polyether polyols with the amines and TDA, the final degradation material was divided into two layers [36,37], where the upper layer was comprised of polyether polyols and the lower layer was comprised of amines, TDA, and other substances. In the preparation process for the polyurethane foam, the recovered polyether polyols (upper phase) played an important role, but this study intended to recycle waste polyurethane elastomers. Therefore, recycling as much as possible was what we needed to focus on. In this study, although the product was divided into upper and lower layers, in order to achieve higher recovery rates, we mixed the upper and lower layers evenly before foaming. Therefore, the GPC measured was also a mixture of the upper and lower phases.

**Figure 5:**
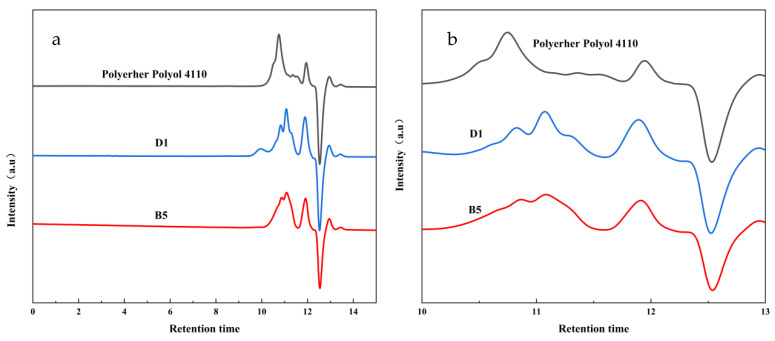
(**a**) Product retention time of polyether 4110 and degradation products D1 and B5 (**b**) Enlarged view of a specific location in (**a**).

As shown in Figure 5, GPC analysis was performed on commercially available polyether 4110 and on the recovered polyether polyol mixtures D1 and B5, respectively. The primary component of commercially available polyether 4110 is polyether polyol, and so it can be seen in the figure that D1, B5, and polyether 4110 had corresponding peaks in a, while the peaks in b appeared in degradation materials D1 and B5. These can be attributed to the amines, TDA, and other substances in the lower phase generated by the byproducts of the high-temperature degradation.

### 3.6. Foaming Time of the Regenerated PU Rigid Foam

The preparation methods of polyurethane foam can be divided into one-step foaming and two-step foaming [38]. As the name implies, one-step foaming involves mixing polyether polyols, catalysts (dibutyltin dilaurate and triethanolamine) [39,40], foaming agents, surfactants, and black materials and stirring, and the reaction and foaming occur at the same time. The hydroxyl in the polyether polyol and the isocyanate in the black material can be used to create a chain extension reaction for two-step foaming, which is divided into the prepolymer method and the semi-prepolymer method, and the common point of the two is that all or part of the white material and the black material are mixed to form prepolymer before foaming, and then other additives are added, as is another part of the mixture of the black material and white material.

**Figure 6:**
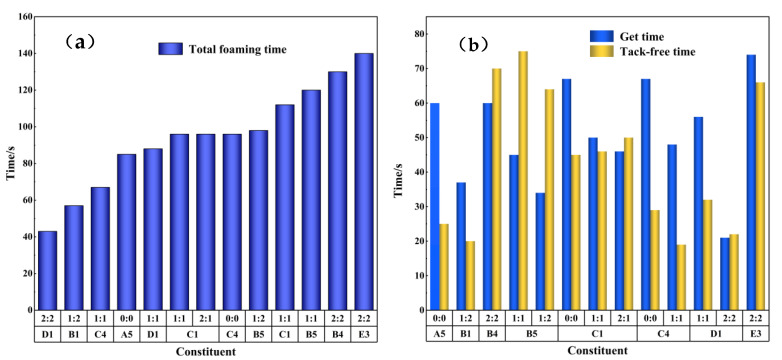
Comparison of the total foaming time (**a**) and the milky white time and gel time (**b**) in the foaming process of the regenerated polyurethane rigid foam prepared by the eight groups of optimal degradation products.

The method adopted in this paper was one-step foaming, and the catalyst was the most direct factor affecting foaming time. Generally, amine and tin compounds are used as catalysts for polyurethane foaming. According to the synergistic effect between the two catalysts, the milky white time and gel time in the foaming process of the degradation material obtained from the degradation of the polyurethane elastomer were roughly measured using the simple industrial empirical method, and the time range obtained is shown in Figure 6.

It can be seen from the figure that the total foaming time of the selected components was between 40–140 s. Generally speaking, within a certain range, with an increase in catalyst addition, the foaming time will be actively shortened [41]. However, the experimental results in this paper were obviously inconsistent with this conclusion because the polyurethane elastomer used in this paper was industrial waste. Although the waste had been cleaned, to a certain extent, before undergoing degradation, during the glycolysis process [42], the metals attached to or wrapped in the waste turned into metal ions in the alcoholysis agent at high temperatures. These metal ions also played a catalytic role in the foaming process. Thus, the final knot was skewed by the presence of metal ions. This was also the primary reason why the foaming time of the components with triethanolamine and dibutyltin dilaurate was shorter than that of the components with the catalyst.

In actual production, the uses of polyurethane foam are different, and the requirements for foaming time are also different. For polyurethane foam wrapped in directly buried pipe, it is hoped that the foaming time is relatively longer and the fluidity is better because of the pipe’s length. For wall-spraying polyurethane foam, the foaming time is attempted to be as short as possible for faster forming.

### 3.7. Water Absorption of the Regenerated PU Rigid Foam

**Figure 7:**
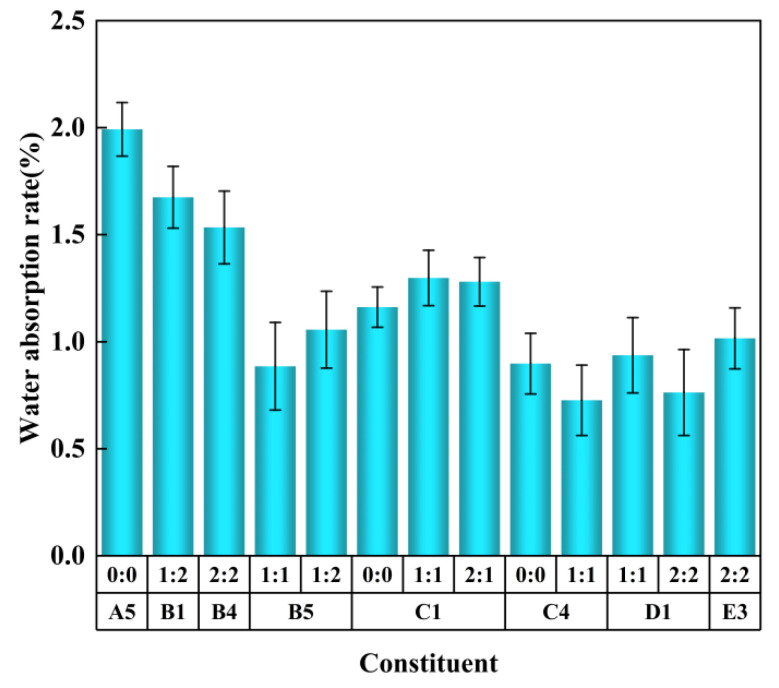
Determination of the water absorption of the regenerated polyurethane rigid foam prepared by the eight groups of optimal degradation products.

Figure 7 shows the water absorption measurement data of the regenerated polyurethane rigid foam prepared by the one-step foaming of the selected eight groups of degradation products. In the foaming process for each group of degradation materials, the ratios of amine to tin varied (0:0, 1:1, 1:2, 2:1, 2:2) so as to prepare five groups of regenerated polyurethane foam. The prepared five groups of regenerated polyurethane foam were cut into pieces for sample preparation, and the water absorption of each group of foam was determined so as to select the regenerated polyurethane rigid foam that best met the standards.

It can be clearly seen in Figure 7 that the water absorption rate of the optimal regenerated polyurethane foam was no more than 0.6%, and this can be used to judge the degree of density between the holes of the polyurethane foam. The most direct factor affecting density is the molecular weight of the polyether polyol and the proportion of isocyanate added. Generally speaking, reducing the molecular weight of polyether polyol or increasing the proportion of isocyanate can improve the density of foam and reduce foam water absorption. The lower the water absorption rate, the more complete the internal bubble structure of the regenerated polyurethane foam, the closer the fit between the bubble and the bubble, and the higher the uniformity of the bubble and the obturator rate [43]. This indicates that the prepared regenerated polyurethane foam had good air tightness and a good thermal insulation effect.

### 3.8. Determination of Density, Strength, and Thermal Conductivity of the Recycled PU Hard Foam

**Figure 8:**
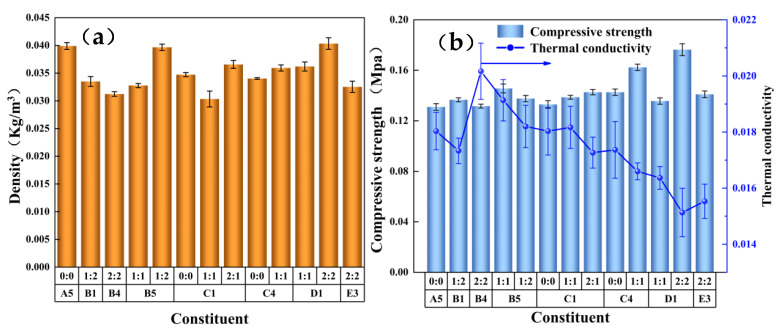
Determination of (**a**) foam density and (**b**) compression strength and thermal conductivity of the regenerated polyurethane rigid foam prepared by the eight groups of optimal degradation products.

As an important performance reference index of polyurethane foam, the apparent density directly affects its compression strength and thermal conductivity. Figure 8 shows the density, compressive strength, and thermal conductivity of the regenerated polyurethane foam of the preferred component. As can be seen in the figure, the density of the regenerated polyurethane foam had a similar variation trend in its compressive strength, and the compressive strength changed with the density. The apparent density of the optimized regenerated polyurethane foam ranged from 30 kg/m^3^ to 40 kg/m^3^, which was consistent with the range of low-density polyurethane foam. The range of compressive strength was 0.13–0.18 MPa, the highest thermal conductivity was 0.0192 W (m·K) ^−1^, and the lowest thermal conductivity was 0.0132 W (m·K)^−1^.

### 3.9. SEM Analysis of the Regenerated PU Rigid Foam

**Figure 9:**
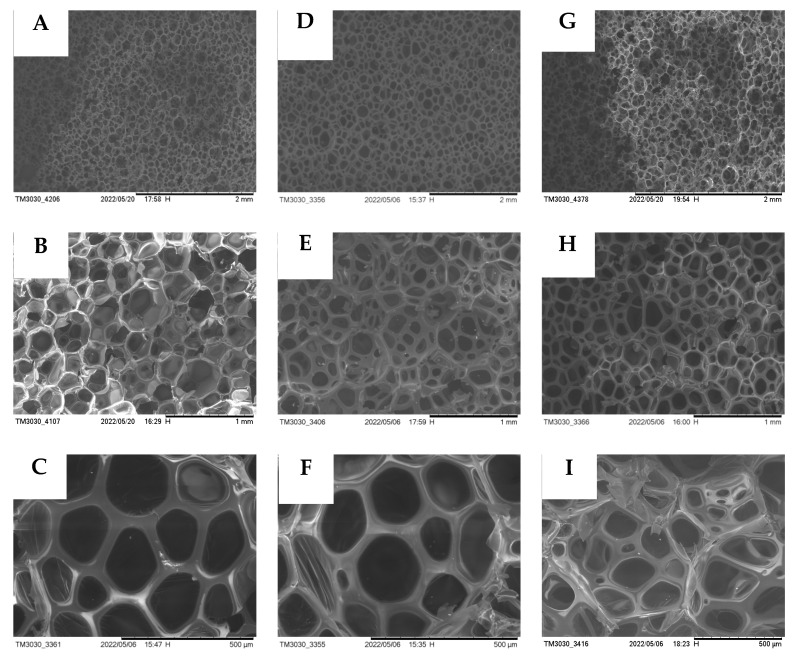
The SEM images of D1 and B5 of the optimal degradation products at three different sizes (2 mm, 1 mm, and 500 μm) were compared with those of polyether 4110. (**A**–**C**) show polyether 4110, (**D**–**F**) show D1, and (**G**–**I**) show B5.

The degradation material obtained from the degradation of the waste polyurethane elastomer by alcoholysis was foamed using the one-step method, and the prepared regenerated polyurethane foam was tested by SEM. The test results are shown in Figure 9.

Figure 9 compares the foams prepared by two types of commercially available polyether 4110 from the eight optimal products. A–C are the electron microscopic images of the foam prepared by commercially available polyether 4110 at different magnifications, and A is the electron microscopic image of the polyurethane foam prepared by commercially available polyether 4110 at different sizes (2 mm, 1 mm, and 500 μm). B is the electron microscopy of the regenerated polyurethane foam prepared by polyether polyols degraded by an alcoholysis agent (DEG: BDO) at different sizes (2 mm, 1 mm, and 500 μm). C is the electron microscopy of the regenerated polyurethane foam prepared by polyether polyols degraded by an alcoholysis agent (EG: BDO) at different sizes (2 mm, 1 mm, and 500 μm). It can be seen that the micromorphology of the regenerated polyurethane foam prepared by the alcoholysis method is similar to the foam prepared by the commercially available polyether 4110 in terms of the integrity of the bubble holes and the strength of the skeletons. Therefore, the alcoholysis method can successfully degrade the waste polyurethane elastomer and prepare regenerated polyurethane foam.

**Figure 10:**
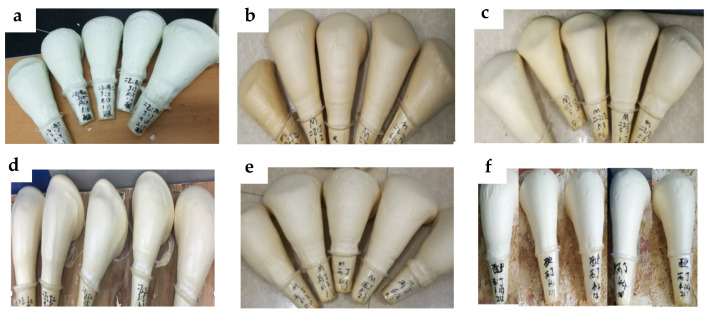

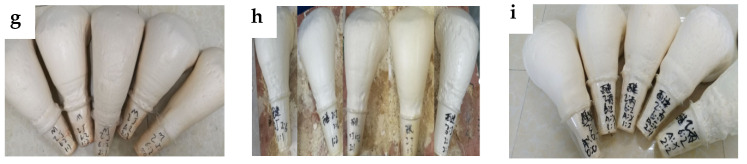
Actual picture of the polyurethane rigid foam partially prepared in the experiment ((**a**–**i**) are regenerated polyurethane rigid foam prepared by degradation products).

Under SEM, the foam holes of the regenerated polyurethane foam showed a uniform “honeycomb” shape [44]. Under the SEM with a size of 2 mm, the bubble holes were tightly and evenly arranged in the visible field of vision, and it could be intuitively observed that the degradation material recovered from degradation could be used as a substitute for polyether polyol to foam and to prepare the regenerated polyurethane foam. From the figure at 500 μm, it can be clearly seen that the shape of the bubble presented regular pentagonal and hexagonal geometric shapes, the skeleton was strong, and the connections between the bubbles were tight, which indicated that the regenerated polyurethane foam prepared using the degradation material of the waste polyurethane elastomer recovered by the alcoholysis method had good sealing properties. The thick skeleton indicated that the foam had a strong compression performance. Under SEM, the test also showed that the bubble holes prepared using the degradation material displayed similar phenomena to the bubble holes prepared using polyether 4110. The tight connection between the bubble holes indicated that the foam had good air tightness and a good thermal insulation performance. Figure 10 shows physical pictures of the regenerated polyurethane foam prepared by one-step foaming in the experiment. It can be clearly seen in the pictures that the regenerated polyurethane foam freely foamed by one-step foaming had smooth surfaces, complete cross-linking, and no slag loss.

### 3.10. TG Analysis of the Regenerated PU Rigid Foam

**Figure 11:**
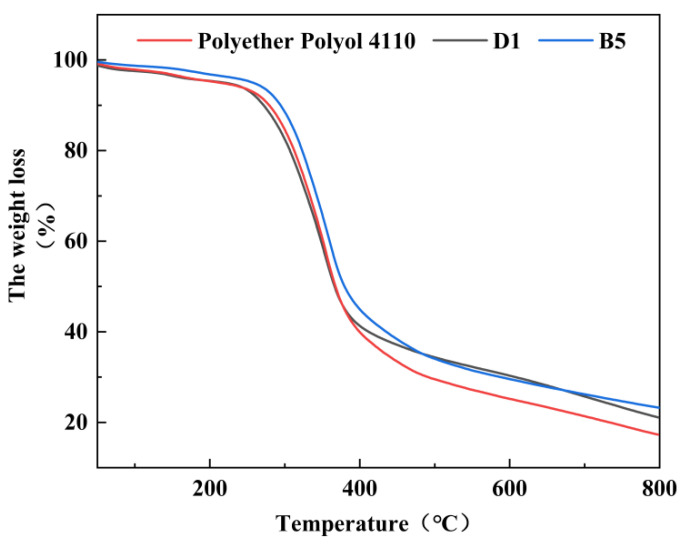
Thermogravimetric diagram of the hard foam of the regenerated polyurethane prepared using polyether 4110 and the D1 and B5 degradation materials.

Figure 11 shows the thermogravimetric analysis of the regenerated polyurethane rigid foam prepared using polyether 4110 and the regenerated polyurethane rigid foam prepared using the degradation materials D1 and B5 and obtained by recycling the waste polyurethane elastomer.

As can be seen from the figure, the thermal weight loss of the two foams with different ratios could be divided into three stages. The first stage was 100–200 °C, and the volatilization of the free and bound water in the foam led to its weight loss. The second stage was 200–390 °C, during which the isocyanic acid in the polyurethane joint segment broke with the hard segment. At this stage, the polyether satin in the end of the polyurethane chain broke [45]. The mass loss temperature of the foam prepared by the two ratios was approximately 250 °C, and the decomposition stopped at approximately 780 °C. Finally, the fastest weight loss temperature of polyether 4110 and D1 was approximately 338 °C, while the fastest weight loss temperature of B5 was approximately 350 °C. It was obvious that the weight loss speeds of polyether 4110 and D1 were significantly faster than that of B5. This indicated that the chemical bond energy of the foam prepared by degradation material B5 was greater, and the human stability of the regenerated polyurethane foam prepared by the degradation material recovered from the waste polyurethane elastomer was similar, or even better, than that of polyether 4110. Therefore, it was shown that the thermal stability of the recycled polyurethane foam prepared by recycling and degradation was more stable, and the strain capacity of the polyurethane foam to environmental changes had improved in the actual use process.

## 4. Conclusions

In this paper, four kinds of diols (butanediol, propylene glycol, ethylene glycol, and monodiethylene glycol) were used to degrade waste polyurethane elastomers successfully, and the regenerated polyether polyol, which was similar to polyether polyol 4110 in its structure and properties, could be used as a raw material for polyurethane synthesis. Eight groups of optimal formulations were selected according to the viscosity of the degraded materials and the compression strength, density, water absorption, and thermal conductivity of the regenerated polyurethane rigid foam. The indexes of the products of the optimal formulations were all within the range of industry requirements, and the viscosity of the recovered degraded materials was between 485 and 1200 MPa·s. The regenerated polyurethane rigid foam was prepared by using the degradation materials rather than commercially available polyether polyols, and the foaming time of the regenerated polyurethane rigid foam was generally between 40 and 140 s. The compressive strength was between 0.131 and 0.176 MPa, and the water absorption rate ranged from 0.7265 to 1.9923%. The apparent density of the foam was between 0.0303 and 0.0403 kg/m^3^. The thermal conductivity ranged from 0.0151 to 0.0202 W/(m·K). After synthesizing all the data, the optimal group was divided into D1: DEG:BDO = 40:40. The compressive strength, water absorption, thermal conductivity, and foam density of the recycled polyurethane foam prepared from this component were the best. From the above data, it can be seen that the recovery of waste polyurethane elastomers by the alcoholysis method and different proportions of alcoholysis agents was successful, and this has certain practical significance for the selection of alcohol types and proportions in later stages.

## Figures and Tables

**Table 1:** Raw materials of the paper experiment.

Reagent Name	The Purity	Factory of Production
Waste polyurethane elastomer	Industrial waste	Shanghai Hecheng Polymer Material Co., Ltd.
Ethylene glycol (EG)	AR	Tianjin Chemical Reagent Factory 1
1,2-Propanediol (PG)	AR	Tianjin Chemical Reagent Factory 1
Butane-1,4-diol (BDO)	AR	Tianjin Kaitong Chemical Reagent Co., Ltd.
Diethylene glycol (DEG)	AR	China Pharmaceutical Guangzhou Chemical Reagent Company
Potassium hydroxide (KOH)	AR	Tianjin Tianli Chemical Reagent Co., Ltd.
Silicone oil stabilizer	CP	Guangzhou Feirui Chemical Co., LRD
TEA	AR	Shanghai Demao Chemical Co., Ltd.
Dibutyltin dilaurate	AR	Shanghai Jieer Technology Co., Ltd.
Foaming agent	CP	Shenzhen Huachang Chemical Co., Ltd.
Polyether 4110	CP	Shandong Lianhaoyao New Material Co., Ltd.
Polyaryl polymethylene isocyanate (PAPI)	CP	Wuhan Fude Chemical Co., Ltd.

Note: Deionized water was used in all of the experiments.

**Table 2:** Experimental equipment used in the paper.

Name of Instrument	Model	Factory of Production
Electronic analytical balance	JA3003C	Sartorius Scientific Instruments (Beijing) Co., Ltd.
Cantilever constant speed power electric mixer	TJ-1200W	Changzhou Huaao Instrument Manufacturing Co., Ltd.
Spherical reactor (1 L)	ZNHW-200	Shanghai Leighton Industrial Co., Ltd
Digital blast drying oven	WX881	Wujiang Weixin Electric Heating Equipment Co., Ltd.
Digital viscometer	NDJ-5	Shanghai Pingxuan Scientific Instrument Co., Ltd.
Disposable plastic cup	350 mL	Topu Daily Chemicals (China) Co., Ltd.
Constant temperature heating sleeve	FDSG-420	Wuxi Huachang Chemical Co., Ltd.

**Table 3:** Statistical table of the best component degradation materials of the eight groups of alcoholysis agents.

Number	Proportion	Viscosity (mPa·s)	Proportion of Catalyst	Wire Drawing Time (s)	Debonding Time (s)
A5	50:30	605.1	-	60	25
B1	40:40	992.4	1:2	37	20
B4	20:60	692.5	2:2	60	70
B5	60:20	660.2	1:1	45	75
			1:2	34	64
C1	40:40	750.6	-	67	45
			1:1	50	46
			2:1	46	50
C4	60:20	555.0	-	67	29
			1:1	48	19
D1	40:40	1208.4	1:1	56	32
			2:2	21	22
E3	60:20	484.2	2:2	74	66

**Table 4:** Statistical table of the hard foam data of the eight groups of alcoholysis agents with the best components.

Number	Proportion	Density (Kg·m^−3^)	Water Absorption Rate (%)	Strength of Compression (MPa)	Coefficient of Thermal Conductivity (W·(m·K)^−1^)
A5	50:30	0.0399	1.9923	0.131	0.0180
B1	40:40	0.0335	1.6748	0.137	0.0173
B4	20:60	0.0312	1.5339	0.132	0.0202
B5	60:20	0.0328	0.8853	0.146	0.0191
		0.0397	1.0561	0.138	0.0182
C1	40:40	0.0347	1.1618	0.133	0.0180
		0.0303	1.2980	0.139	0.0182
		0.0366	1.2802	0.143	0.0173
C4	60:20	0.0340	0.8976	0.143	0.0174
		0.0359	0.7265	0.162	0.0166
D1	40:40	0.0362	0.9368	0.136	0.0164
		0.0403	0.7630	0.176	0.0151
E3	60:20	0.0325	1.0157	0.141	0.0155

## Data Availability

Not applicable.
